# Exosomes and cancer immunotherapy: A review of recent cancer research

**DOI:** 10.3389/fonc.2022.1118101

**Published:** 2023-01-16

**Authors:** Yue Cao, Peng Xu, Yangling Shen, Wei Wu, Min Chen, Fei Wang, Yuandong Zhu, Feng Yan, Weiying Gu, Yan Lin

**Affiliations:** ^1^ Department of Hematology, The Third Affiliated Hospital of Soochow University, Changzhou, Jiangsu, China; ^2^ Department of Hematology, Soochow Hopes Hematology Hospital, Suzhou, Jiangsu, China

**Keywords:** extracellular vesicle, exosome, tumor immune microenvironment, gastrointestinal cancer, immunotherapy

## Abstract

As phospholipid extracellular vesicles (EVs) secreted by various cells, exosomes contain non-coding RNA (ncRNA), mRNA, DNA fragments, lipids, and proteins, which are essential for intercellular communication. Several types of cells can secrete exosomes that contribute to cancer initiation and progression. Cancer cells and the immune microenvironment interact and restrict each other. Tumor-derived exosomes (TDEs) have become essential players in this balance because they carry information from the original cancer cells and express complexes of MHC class I/II epitopes and costimulatory molecules. In the present study, we aimed to identify potential targets for exosome therapy by examining the specific expression and mechanism of exosomes derived from cancer cells. We introduced TDEs and explored their role in different tumor immune microenvironment (TIME), with a particular emphasis on gastrointestinal cancers, before briefly describing the therapeutic strategies of exosomes in cancer immune-related therapy.

## 1 Introduction

The tumorigenesis of cancer can be attributed to the interaction between cells and the tumor immune microenvironment. Normal cells undergo genetic changes upon chemical, biological, and physical stimulations, leading to cell growth and differentiation from an orderly and controllable state to uncontrolled monoclonal proliferation. Meanwhile, the immune system evades (1), stromal cell niche transformation (2), angiogenesis (3), and other changes in the tumor immune microenvironment (TIME), providing permissive conditions for the invasion and development of cancer cells and promoting the occurrence and development of cancers (4). At present, the mainstream regimens of cancer treatment include surgery, radiotherapy, and chemotherapy. However, the treatment effect remains poor due to the lack of specificity, and the side effects are significant, which has been confirmed in related studies on breast cancer (BC) (5, 6), colorectal cancer (CRC) (7–9), lung cancer (10), blood cancer (11, 12), and so on. With the in-depth study on the mechanism of cancerogenesis, targeted therapy provides a feasible scheme to overcome the limitations of traditional treatment approaches (13). Nucleic acids and proteins involved in cancer development both within the tumor cell and in the TIME can be potential targets for targeted therapy (14). At present, targeted therapies targeting human epidermal growth factor receptor 2 (HER-2), vascular endothelial growth factor (VEGF) (15), and tyrosine kinase are widely used in gastrointestinal cancers (16) and hematological cancers (17). However, targeting only these targets within cancer cells sometimes leads to the development of resistance and relapse (18–22). The TIME includes immune cells, fibroblasts, peripheral blood vessels, bone marrow (BM)-derived inflammatory cells, various signaling molecules, and extracellular matrix components. These components interact and communicate with each other to maintain the balance between procancer and anticancer mediators. However, cancers can shape the TIME into an immunosuppressive state conducive to tumor growth and disrupt this balance. Therefore, targeting the TIME, rebalancing the TIME, and restoring the immune self-inspection of the body itself can be considered a more comprehensive targeted therapy strategy with fewer side effects (23, 24).

As a therapeutic strategy, cancer immunotherapy aims to reshape the TIME, restore the cancer-killing function of immune cells, and enable cancer cells to be killed by the autoimmune system (25). Based on the understanding of cancer immune escape, several immunotherapies have been developed to inhibit cancer growth. Ipilimumab, an immune checkpoint cytotoxic T lymphocyte antigen 4 (CTLA-4) inhibitor, has successfully allowed 21% of patients to survive for more than 10 years (26). Programmed death 1 (PD-1) drugs help 30% of patients with the terminal disease survive for more than 5 years (27). An autologous CD19-targeting chimeric antigen receptor CAR T-cell product is approved for treating R/R B-cell acute lymphoblastic leukemia (ALL) and lymphoma (28). These immunotherapy methods provide exciting new options for clinical practice. However, cancer drug resistance and low efficacy suggest that we still have more content to explore for cancer immunotherapy.

**Figure 1:**
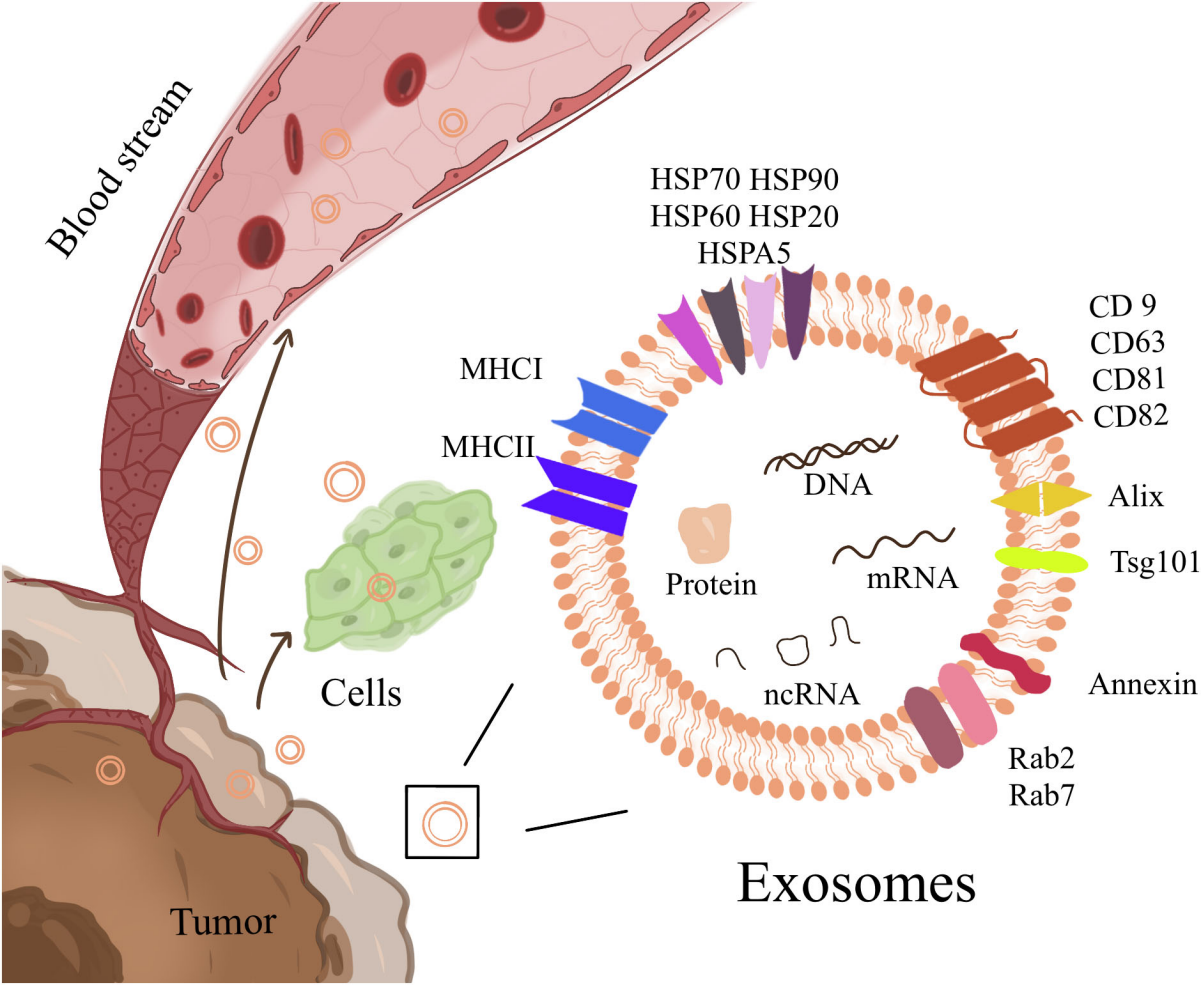
Exosomes are phospholipid extracellular vesicles that range in size from 40nm to 100nm and contain ncRNA, mRNA, DNA fragments, lipids, and proteins, including some conserved proteins and cell-specific biomarkers. It is essential for intercellular communication, transport, and flow in body fluids.

As phospholipid extracellular vesicles (EVs) of approximately 40-100 nm in size, exosomes are secreted by almost all cells, which can contain microRNA (miRNA), mRNA, DNA fragments, lipids, and proteins and are essential substances for intercellular communication (29, 30) (**Figure 1**). These vesicles include some conserved proteins, such as tetraspanins (CD9, CD63, CD81, and CD82), cell membrane fusion and transport proteins (Rab2, Rab7, flotillin, and annexin), heat shock proteins (HSP70, HSP90 Hsp60, and Hsp20), cytoskeleton proteins (β-actin and tubulin), and synthesis proteins (Alix and Tsg101). Among them, CD63/TSG101 is the most commonly used exosome marker (31). Exosomes carry their contents throughout body fluids (blood, saliva, urine, and feces) and interact with target cells through direct fusion (32) or endocytosis (33, 34) to transfer the information to target cells and exert their effects. Studies have shown that tumor-derived exosomes (TDEs) can act on target cells to promote cancer angiogenesis (35, 36), facilitate the transformation of cancer stromal cells to a pro-cancer phenotype, and change the cancer niche (2, 37) to urge cancer initiation and progression. In clinical data statistics, the molecular content of exosomes has an excellent prognostic or predictive value in cancer progression and treatment response of cancer patients (38). Exosomes also include specific biomarkers of their cellular origin. Examples include costimulatory molecules derived from immune cells, such as B cells and dendritic cells (DCs) (39–41), and exosomes derived from cancer cells, which contain cancer-specific antigens (42), playing a vital role in cancer immune presentation and cancer-specific immune responses. Therefore, the research and application of exosomes in immunotherapy have attracted much attention (43). It was shown 20 years ago that vaccination with DCexos, derived from BM-derived DCs, has better anticancer efficacy than vaccination with DCs, supporting the development of cancer vaccines (44). Inhibition of exosomal PD-1 reduces resistance to PD-1 therapy (45). Here, we summarized the current knowledge regarding exosomes in cancers and the TIME, focusing on gastrointestinal cancers, and discussed their potential use in immunotherapy. Using this strategy, we can approach the enhancement of cancer immunotherapy from a more scientific perspective.

## 2 Discovery of exosomes and their roles in cancer progression

### 2.1 Effects of non-TDEs on cancers

Various cells can secrete exosomes. Han et al. have found that exosomes containing *miR-145* secreted by tumor-associated stroma in pancreatic ductal adenocarcinoma can act on adjacent pancreatic ductal adenocarcinoma cells to inhibit cancer cell growth (46). Cancer-associated fibroblasts (CAFs) are the main components of the TIME. Studies have found that exosomes secreted by CAFs transmit information, such as *miR-181D-5P*, *miR-21*, *miR-143*, and *miR-378e*, to BC cells, which promote stem cell characteristics, epithelial-mesenchymal transition (EMT) phenotype, and non-anchoring cell growth of BC cells to encourage the progression of cancer (47–49). CAFs can also secrete exosomes carrying *SNHG17* to mediate osteosarcoma proliferation and metastasis (50). Adipocytes also secrete exosomes, leading to increased cancer cell migration and invasion (51). Mesenchymal stem cells (MSCs) also exist in the TIME and have immunomodulatory properties. Mao et al. have shown that BM-MSCs from *p53*-deficient mice (*p53* mBM-MSCs) can regulate the Wnt/β-catenin pathway and deliver *UBr2* to target cells, thereby promoting the growth and metastasis of gastric cancer (GC) (52). Ma et al. have found that exosomes derived from BM-MSCs transfected with *miR-221* significantly promote the proliferation, migration, invasion, and adhesion of GC BGC-823 and SGC-7901 cells (53). Human BM-MSC exosomal *miR-375* inhibits glioma cell progression through *solute carrier family 31 member 1(SLC31A1*)inhibition (54).

Immune cell-derived exosomes in the tumor microenvironment also play an important role in the occurrence and development of cancers. As early as 2012, Luana et al. have proposed an important role of natural killer (NK) cell-derived exosomes in immune surveillance and homeostasis (55). Aladin and colleagues showed that NK cell-derived exosomes eliminated leukemia cells isolated from patients with leukemia (56). Zitvogel’s earlier study showed that DC cell-derived exosomes were as effective as DC cells in inducing tumor immune responses, which also provided theoretical support for cancer vaccine research (44). Romagnoli et al. indicated that breast cancer cells treated with DC cell-derived exosomes were more effective in stimulating T cell responses than breast cancer cells without exosome treatment (57). Cai and colleagues showed that CD8+ T cell-derived Exos can promote tumor cell invasion and lung metastasis through the Fas pathway (58).Xie et al. have demonstrated that Natural CD8+25+ regulatory T cell-secreted exosomes can inhibit CD8+ T cell responses and antitumor immunity (59). Wang et al. have shown that exosomes derived from Tumor-associated macrophages up-regulate *miR-192-5p* to promote endometrial cancer cells apoptosis and effectively suppress the progression of endometrial cancer (60). Exosomes secreted by polymorphonuclear (PMN) myeloid-derived suppressor cells (MDSCs), which are immunomodulatory cells present in the TIME, significantly inhibit NK cell-mediated anticancer activity, suggesting that the detection of PMN-MDSC content may have prognostic value (61). *miR-326* in M1 macrophage exosomes promotes apoptosis of liver cancer cells and inhibits cancer proliferation and migration by downregulating the expression of NF-κB in liver cancer (62).

Cancers interact with various cells in their TIME. In the above review, we briefly discuss the role of non-tumor derived exosomes in tumors, next, we focused on the role of TDEs.

### 2.2 Effects of TDEs on cancers

TDEs function in both autocrine and paracrine ways in the TIME. They can act on the cancer cells themselves or on the surrounding cells and tissues to affect cancer development, which has been confirmed in various cancers (**Table 1**). Furthermore, TDEs can affect other non-cancer cells and participate in the transfer of metastatic capacity. For example, exosomes released by malignant BC cells are taken up by less malignant cancer cells located within the same cancer, thereby promoting the migration and metastatic potential of low-grade malignant cancer cells (100). In addition, TDEs can act on immune cells in the microenvironment to regulate cancer immunity and affect the balance between cancer promotion and cancer suppression in the TIME.

**Table 1:** Roles of various tumor-derived exosomes in tumors.

Cancer Type	Origin of Exosome	Biomarker	Pathway/Targets	Recipient Cells	Results(*in vitro*)	Effect on TIME	Results(*in vivo*)	Effect on tumor progression	Ref
Gastric cancer	MGC-803, MKN-45	*LSD1*		MGC-803, MKN-45	Increased *Nanog*, *OCT4*, *SOX2* and CD44 expression to increase stemness of cancer cells		Promoted tumor growth	Up	(63)
Colon cancer	SW480	*ADAM17*	E-cadherin	SW480	Cleaved the E-cadherin		Promoted liver metastasis of tumor	Up	(64)
Gastric cancer	XGC-1 cell, MKN45, GES1	*CircNEK9*	*miR-409-3p/MAP7* Axis	XGC-1 cell, MKN45, GES1			Promoted proliferation,metastasis and invasion of tumor	Up	(65)
Gastric cancer	HGC-27, AGS, GES-1, MGC-803, KATOIII, MKN45, BGC-823	*circ-RanGAP1*	*miR-877-3p/VEGFA*	HGC-27, AGS, GES-1, MGC-803, KATOIII, MKN45, BGC-823			Promoted metastasis and invasion of tumor	Up	(66)
Colon cancer	DLD1, SW480, HCT8	*CircCOG2*	miR-1305/TGF-β2/SMAD3 pathway	DLD1, SW480, HCT8			Promoted proliferation,metastasis and invasion of tumor	Up	(67)
Gastric cancer	SGC7901/ADR cell(anti-drug resistance)	*miR-501*	*BLID*/caspase-9/-3	SGC7901			Promoted tumor resistance to doxorubicin and promoted proliferation,metastasis and invasion of tumor	Up	(68)
Gastric cancer	GES-1, BGC-823, OCUM-1	*miR-675-3p*	*CXXC4*	GES-1, BGC-823, OCUM-	Up-regulation of MAPK/PD-L1 and inhibited immunity	Down	Promoted immune escape	Up	(69)
Gastric cancer	MGC-803	*miR-1290*	Grhl2/ZEB1/PD-L1 axis	MGC-803	Up-regulation of PD-L1 and inhibited Tcell immunity	Down	Promoted immune escape	Up	(70)
Colon cancer	KM12SM	*miR-106b-3p*	*DLC-1*	SW480	Promoted metastasis, invasion and EMT of tumor		Promoted lung metastasis of tumor	Up	(71)
Colon cancer	SW620	*miR-335-5p*	*RASA1*	SW620	Promoted EMT		Promoted metastasis, invasion of tumor	Up	(72)
Gastric cancer	MKN45	*FRLnc1*		MKN45			Promoted proliferation,metastasis of tumor	Up	(73)
Colon cancer	SW480, SW1463, HT-29	*lncRNA KCNQ1OT1*	*miR-30a-5p/USP22*	SW1463	Regulated PD-L1 ubiquitination and inhibited CD8+T cell immunity	Down	Promoted immune escape	Up	(74)
Gastric cancer	MKN-28,MKN-45, SGC-7901			CD4+T cells, MDSC, CD8+T cells, and NK cells	Promoted the apoptosis of CD8+T cells	Down	Promoted lung metastasis of cancer cells	Up	(75)
Gastric cancer	SGC7901, MGC803, MKN45	*miR-135B-5p*	*miR-135B-5p*/sp1 axis	V-γ-9V-δ-2T cell	Induced apoptosis of V-γ-9V-δ-2T cel and reduced production of IFN-γ and TNF-α	Down	Promoted immune escape	Up	(76)
Gastric cancer	BGC8-23,MGC80-3, SGC-7901		*HMGB1/TLR4*/NF-κB	Neutrophils	Induced neutrophil autophagy and promoted tumor growth	Down	Promoted tumor growth	Up	(77)
Gastric cancer	BGC-823	*HMGB1*	*STAT3*	Neutrophils	Promoted PD-L1 expression of Neutrophils	Down	Promoted immune escape	Up	(78)
Epstein‐Barr virus-associated GC	SNU719, MKN7+EBV, MKN74 + EBV			DCs	Inhibitied of DC maturation	Down	Promoted immune escape	Up	(79)
Colon cancer	RKO, CaR-1	*miR-203*		Macrophages	Pushed macrophage polarization toward M2 phenotype		Promoted immune escape	Up	(80)
Gastric cancer	MGC-803, BGC-823, SGC-7901	*miR-107*	*DICER1, PTEN*	MDSCs	Inducted aggregation and expansion of MDSCs	Down	Promoted immune escape	Up	(81)
Gastric cancer		TGF-β1		Treg cell	Induced Treg cells formation	Down	Promoted tumor lymph node metastasis	Up	(82)
Colon cancer	HCT116, SW620	*HSPC111*		CAFs	Promoted EMT		Promoted metastasis of cancer cells		(83)
Colon cancer	HCT116, HT29	*miR-1246*	Akt/mTOR/STAT3 axis	Macrophages	Pushed macrophage polarization toward M2 phenotype		Promoted 5-fluorouracil resistance, migration, and tumor sphere formation.	Up	(84)
Gastric cancer	44As3	*miR-193b*		iNF-58, iNF-60	Promoted the differentiation of fibroblasts into chemokines producing fibroblasts		Promoted tumor growth	Up	(85)
Gastric cancer		*miR-519a-3p*		Kupffer cell			Promoted liver metastasis of cancer cells	Up	(86)
Gastric cancer				vascular endothelial cell	Destroyed the brain blood barrier		Promoted metastasis of tumor	Up	(87)
colon cancer	CT26	*IRF-2*		Macrophages	Induced the release of VEGFC from macrophages, induced lymphatic endothelial formation		Promoted tumor lymph node metastasis	Up	(88)
Gastric cancer	SGC7901	ciRS-1*33(hsa_circ_0010522)*	*miR-133/PRDM16*	3T3-L1	Induce preadipocytes’ differentiation into brown fat		Contributed to cancer *cachexia*		(89)
Gastric cancer	SGC7901, MGC803	*miR-155*	C/EPBβ	A-MSC	Induce MSCs’ differentiation into brown fat		Contributed to cancer *cachexia*		(90)
Gastric cancer		*miR-106a*	Smad7, TIMP2/TGF-β	PMC	Promoted apoptosis of PMCS		Promoted peritoneal metastasis of cancer		(91)
Gastric cancer	GC9811-P	*miR-486-5p*		HMRSV5	Down-regulation of *miR-486-5p* promoted the reduction of E-cadherin expression in HMRSV5 cells		Down-regulation of miR-486-5p promoted peritoneal metastasis of cancer	Down	(92)
Leukemia	JM1, SUP-B15	*miR-181a*		JM1, SUP-B15	Up-regulated survival genes and down-regulated apoptotic genes		Promoted tumor cell proliferation	Up	(30)
Leukemia	L1210	TGF-β1		DCs, CD4+T, Th1, NK	Inhibit the cytotoxicity of CTL and NK	Down	Promoted immune escape	Up	(93, 94)
Lymphoma	OCI-LY3, SU-DHL2, and GCB subtype OCI-LY1, SU-DHL8		*GP130/STAT3*	Macrophages	Pushed macrophage polarization toward M2 phenotype		Promoted immune escape		(95)
Lymphoma	WSU-NHL A20	*miR-7e-5p*	FasL- caspase	Macrophages	Promoted the apoptosis of M1 macrophages	Down	Promoted the transformation of tumors to a higher malignant degree	Up	(96)
Lymphoma	WSU-DLCL2、WSU-FSCCL	*survivin*	IFN-γ	NK, CTL	Decreased *NKG2D* levels	Down	Promoted immune escape	Up	(97)
Leukemia	K562	*miR-92a*		Human umbilical vein endothelial cells	Enhanced endothelial cell migration and tube formation		Promoted metastasis of tumor	Up	(98)
Leukemia	KG1A, NB4, and MV411			BMSCs	Promoted the production of IL-8 by BMSCs		Protected the leukemia cells against chemotherapy.	Up	(99)
Breast cancer	4T1	*miR-200*	E-cadherin	4TO7	Promoted EMT		Promoted metastasis of tumor	Up	(100)
Breast cancer	ZR-75-1, MCF-7	*PTPRO*	*STAT*	Macrophages	Pushed macrophage polarization toward M1 phenotype		Inhibited the metastasis and invasion of tumor	Down	(101)
Breast cancer	C57BL/6 EO771	*Gp130*	*STAT3*	Macrophages	Pushed macrophage polarization toward M2 phenotype			Up	(102)
Breast cancer	MDA-MB-231	*miR-105*		MCF-10A	Downregulates Tight Junctions and Destroys the Barrier Function of Endothelial Monolayers		Promoted metastasis of tumors	Up	(36)
Breast cancer	MDA-MB-231, MCF-10A	*miR-20a-5p*	*SRCIN*	MDA-MB-231, BMMS	Promoted the migration and invasion of MDA-MB-231 cells; promoted the proliferation and differentiation of osteoclasts		Promoted bone metastasis of tumors	Up	(103)
Triple-negative breast cancer		PD-L1	*TBK1/STAT6*, *AKT/mTOR*	CD8+T, Macrophages	Inhibited T cell function; Pushed macrophage polarization toward M2 phenotype	Down	Promoted immune escape	Up	(104)
Prostate cancer	LNCAP	*miR-26a*		mCRPC			Inhibited the proliferation and invasive properties of tumor cells	Down	(105)
Prostate cancer	PC3, CWR22Pc	αvβ3		BPH-1, C4-2B	Changed cell phenotype and enhanced tumor cell adhesion and migration		Promoted tumor metastasis	Up	(106)
Prostate cancer	RM-1		TLR2/NF-κB-CXCR4- cxcl12 axis	MDSCs	Promoted the migration of MDSCs into the tumor microenvironment	Down		Up	(107)
Prostate cancer	PC3	*miR-1275*	*SIRT2/Runx2*	hFOB1.19	Increased the activity of osteoblasts		Promote bone metastasis of tumor	Up	(108)
Glioma	Wi38	*SEMA7A*	Integrin β1	GSC			Promoted cell migration	Up	(109)
High Grade Gliomas	HEB、U87和U251	*circGLIS3 (hsa_circ_0002874)*	*Ezrin*	hBMEC	Modulating Ezrin phosphorylation and promoted the angiogenesis of hBMEC.		Promoted vascular metastasis.	Up	(110)
Glioma	GBM	*CD73*	AMP-A2AR- Glycolysis	T-cell	Inhibit T cell aerobic glycolysis	Down		Up	(111)
Glioblastoma		*lncRNA SBF2-AS1*	*XRCC4*	GBM cells			Enhanced chemoresistance to temozolomide in glioblastoma.	Up	(112)
Lung cancer	A549, NCI-H23, NCI-H1299, and HCC827	*miR-2682-5p*	*HDAC1/ADH1A* axis	A549, NCI-H23, NCI-H1299, and HCC827	Inhibited cell migration and promote apoptosis		Promoted tumor growth	Up	(113)
Lung cancer	NCI-H1299、A549	*circUSP7*	*miR-934/SHP2* axis	CD8+T cell	Inhibited the secretion of IFN-γ, TNF-α, granzyme B and perforin by CD8+T cells。	Down	Promoted immune escape	Up	(114)
Lung cancer		*circPVT1*	*miR-124-3p/EZH2* axis	Macrophages	Pushed macrophage polarization toward M2 phenotype		Promoted proliferation,metastasis and invasion of tumor	Up	(115)
Salivary Gland Adenoid Cystic Carcinoma	SACC-83		*Claudins, ZO-1*	SACC-83, HUVEC	Increased cell inner membrane permeability		Promoted, metastasis of tumor	Up	(116)
Uveal melanoma			*MIF*			Down	Promoted, metastasis of tumor	Up	(117)
Nasopharyngeal carcinoma	CNE-1, CNE-2,5-8F,6-10B	*MIF*		Macrophages	Inhibited ferroptosis and promotes M2 polarization in macrophages		Promoted tumor growth	Up	(118)
Clear Cell Renal Cell Carcinoma	ccRCC cells	TGF-β1	TGF-β/*SMAD*	NK cell	Induced NK cell dysfunction	Down	Promoted immune escape	Up	(119)

#### 2.2.1 Gastroenteric cancer

Gastrointestinal cancer is the fourth most deadly cancer in the world (120). Despite great progress in surgery combined with radiotherapy and chemotherapy, its early diagnosis and late prognosis are still noteworthy (121). Tumor-derived exosomes are early participants in the tumor’s participation in the immunosuppressive microenvironment, which in turn affects the survival and treatment response of gastric cancer patients (122). Exploring the mechanism of gastrointestinal cancer-derived exosomes on tumor immune microenvironment will provide more targets for diagnosis and treatment.

**Figure 2:**
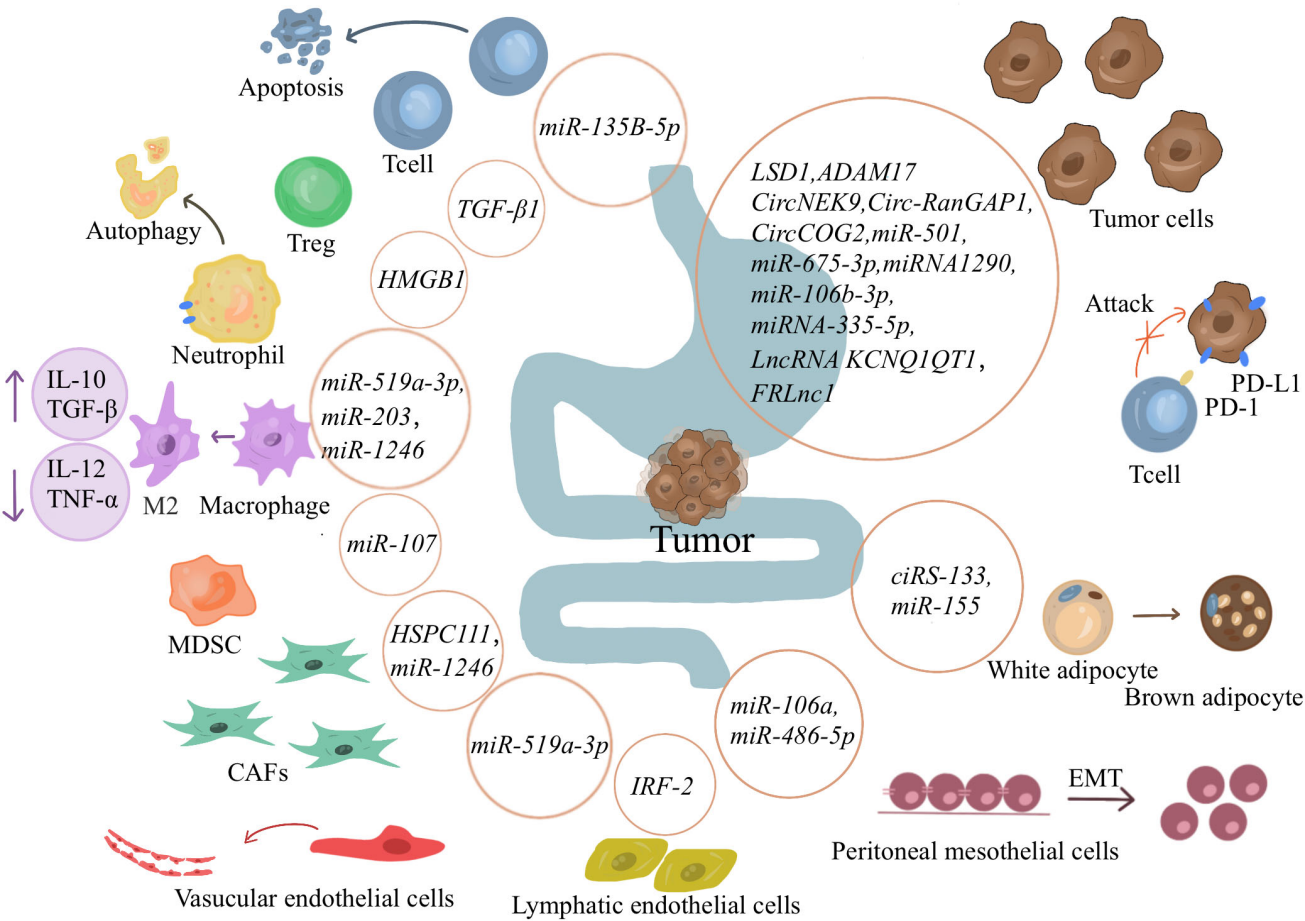
Exosomes’ role in gastrointestinal tumors. Cancer-derived exosomes can promote tumor proliferation, metastasis, and invasion by transferring information such as proteins, circRNA, miRNA, and lncRNA to tumor cells. It can also affect immune cells, causing an immunosuppressive environment and promoting tumor immune escape. Exosomes also affect other cells in the immune microenvironment and other tissue cells, directing them in a more tumor-friendly direction. And encourage tumor cells to spread to distant locations.

Gastrointestinal TDEs play a vital role in gastrointestinal cancerogenesis. TDEs can act on cancer cells to promote cancer proliferation, metastasis, and invasion by transferring information, such as proteins, circular RNAs (circRNA), miRNA, lncRNA (**Figure 2**). TEDs delivery proteins affect GC cells.Zhao et al. have reported that *lysine-specific demethylase 1 (LSD1)* in gastric TDEs can act on receptor GC cells to promote cancer stemness (63). Sun and colleagues have revealed that *ADAM17* in TDEs can cleave the E-cadherin junction to promote cancer cell metastasis (64). TEDs delivery circRNA affect GC cells. Overall, circRNAs usually affect the expressions of downstream genes by acting as miRNA sponges. Li et al. have suggested that *CircRNA NIMA-related kinase 9 (circNEK9; hsa_circ_0032683)* in gastric TDEs promotes the proliferation, migration, and invasion of GC cells through the *miR-409-3p*/*MAP7* axis (65). Lu et al. have shown that the expression of *circ-RanGAP1(hsa_circ_0063526)* is high in GC exosomes and promotes the progression of GC through the *miR-877-3p/VEGFA* axis (66).Gao and coworkers have reported that *circCOG2(hsa_circ_0016866)* in the exosomes of colon cancer cells promotes CRC progression by sponging *miR-1305* to promote the proliferation, migration, and invasion of CRC (67). TEDs delivery miRNA affect GC cells. Liu et al. have indicated that *miR-501* in exosomes derived from GC promotes the proliferation, migration, and invasion of cancer cells through the subsequent inactivation of caspase-9/-3 and phosphorylation of Akt (68). Li and colleagues have revealed that *miR-675-3p* in exosomes binds to the 3ʹ untranslated region of *CXXC4*, downregulates the expression of *CXXC4*, and upregulates MAPK/PD-L1, thereby mediating immunosuppression and increasing immune escape (69). Liang et al. have demonstrated that in a coculture system of EVs and T cells, *miR-1290* can upregulate programmed death ligand 1(PD-L1) through the Grhl2/ZEB1 pathway and promote the inhibitory effect of GC cells on T-cell activation (70). Liu et al. have shown that *miR-106b-3p* in exosomes acts on *DLC-1* to promote EMT, cancer cell migration, and invasion (71). Sun et al. have pointed out that exosomes can deliver *miR-335-5p* to metastatic CRC cells, resulting in the EMT of cancer cells and promoting invasion and metastasis of CRC cells (72). TEDs delivery lncRNA affect GC cells.In a study by Zhang and collaborators, gastric TDEs promote the proliferation and migration of GC cells by transporting *FOXM1 related long noncoding RNA (FRLnc1)* (73). Xian et al. have shown that *lncRNA KCNQ1OT1* contained in exosomes secreted by CRC can increase the expression of PD-L1 protein in cancer cells through the *miR-30a-5p/USP22* pathway and mediate immune escape (74).

Gastrointestinal TDEs can also act on immune cells to induce an immunosuppressive environment and promote cancer proliferation and metastasis. As early as 2005, Huber et al. have shown that human CRC cells induce T-cell death by releasing proapoptotic microbubbles (123), suggesting that the role of exosomes in the TIME should not be underestimated. Liu et al. have suggested that GC cell exosomes change the gene expression and cytokine secretion level of CD8+ T cells to promote the formation of an immunosuppressive microenvironment (75). Li and colleagues have found that *miR-135B-5p* in GC exosomes reduces the survival rate of V-γ-9 V-δ-2T cells and induces cell apoptosis through the *miR-135B-5p/*SP1( specificity protein 1) axis. Reduced production of the cytotoxic cytokines interferon γ (IFN-γ) and tumor necrosis factor α (TNF-α) favors cancer progression (76). In addition, Zhang et al. have indicated that TDEs induce neutrophil autophagy through the *HMGB1*/*TLR4*/NF-κB signaling pathway (77). Shi et al. have shown that exosomal transport of *HMGB1* activates *STAT3*, which upregulates PD-L1 expression on neutrophils at the genetic level and suppresses T-cell immunity (78). Hinata et al. have shown that exosomes from Epstein‐Barr virus-associated GC inhibit DC maturation (79). Takano et al. have reported that CRC serum exosomal *miR-203* promotes CRC liver metastasis by enhancing M2 polarization (80). Ren and coworkers have reported that exosomes secreted by GC can carry *miR-107* to the host MDSCs and induce their expansion and activation by targeting *DICER1* and *PTEN* genes to generate an immunosuppressive microenvironment and promote cancer progression (81). Tang has demonstrated that high expression of exosomal TGF-β1 induces an increase in Tregs in GC-draining LNs, which is involved in cancer lymph node metastasis (82).

Gastrointestinal TDEs also affect other cells in the TIME, making them more suitable for cancer development. Zhang et al. have suggested that *HSPC111* promotes the expression and secretion of *CXCL5* by altering lipid metabolism in CAFs, inducing EMT, and promoting CRC cell migration through the *CXCL5*-*CXCR2* axis (83). Huang and colleagues have found that *miR-1246* is highly expressed in exosomes secreted by colon cancer cells, promotes fibroblast proliferation and M2 polarization through the Akt/mTOR/STAT3 axis, and reduces cancer size *in vivo (* 84). Naito and colleagues have revealed that exosomes secreted by highly metastatic GC cells can differentiate fibroblast lines in the microenvironment into chemokine-producing fibroblasts, acting directly on the TIME to make it suitable for cancer growth (85). Qiu et al. have suggested that *miR-519a-3p* in gastric TDEs activates the MAPK/ERK pathway by targeting *DUSP2*, leading to M2-like polarization, indirectly inducing angiogenesis, and promoting cancer metastasis (86). Wang et al. have reported that gastric TDEs can destroy the vascular endothelial barrier and promote cancer metastasis (87). Sun and coworkers have reported that high expression of *IRF-2* in exosomes can lead to lymphatic vessel endothelium and lymphatic grid formation in sentinel lymph nodes, forming a premetastatic niche (88). Zhang et al. and Liu et al. have shown that one circRNA *(hsa_circ_0010522*) and *miR-155* in exosomes derived from GC cells can act on MSCs to induce their differentiation into brown fat, promote the formation of cancer cachexia, and accelerate the death of patients (89, 90).

Exosomes can also affect other tissue cells and promote the metastasis of cancer cells. Mesenchymal transformation and matrix degeneration are prerequisites for disrupting the mesothelial barrier. Zhu et al. have suggested that *miR-106a* in gastric TDEs can be transferred to peritoneal mesothelial cells. It also activates TGF-β signaling by targeting Smad7 and TIMP2 to induce mesothelial mesenchymal transition and extracellular matrix degeneration, thereby promoting metastasis and dissemination of GC (91). Lin et al. have reported that downregulation of *miR-486-5p* in gastric TDEs promotes peritoneal EMT, thereby accelerating peritoneal metastasis of GC (92).

#### 2.2.2 Hematologic neoplasms

Exosomes are involved in the occurrence and progression of hematological cancers. Exosomal *miR-181a* secreted by pediatric acute lymphoblastic leukemia (PALL) cells can promote cancer development by upregulating proliferation genes and downregulating apoptosis genes, and silencing this gene can inhibit cancer cell proliferation (30).Silencing TGF-β1 in leukemia-derived exosomes can promote CD4+ T cell proliferation and Th1 cytokine secretion, thereby stimulating specific cytotoxic lymphocytes and NK cytotoxicity more effectively and enhancing antileukemic immunity (93, 94). Ling et al. have demonstrated that diffuse large B-cell lymphoma (DLBCL)-derived exosomes can promote the transformation of macrophages into the M2 type (95). Lou and colleagues have found that downregulation of exosomal *miR-7e-5p* induces apoptosis in M1 macrophages, leading to immune surveillance and transformation of follicular lymphoma (FL) (96). Ferguson Bennit et al. and Ling et al. have shown that treatment with exosomes does not significantly alter NK-cell functionality, while extracellular *survivin* decreases *NK group 2D receptor (NKG2D*) levels and the intracellular protein levels of perforin, granzyme B, TNF-α, and IFN-γ (97). In a study by Umezu and collaborators, exosomes derived from the leukemic cell line K562 highly express *miR-92a*, which can promote endothelial cell migration and tube formation (98). Chen et al. have shown that exosomes produced by acute myeloid leukemia (AML) KG1A cells can promote the production of IL-8 by bone marrow stromal cells (BMSCs) to promote leukemia drug resistance (99).

#### 2.2.3 Breast cancer

Exosomes are involved in the occurrence and progression of BC. BC cells with high metastatic capacity can secrete exosomes carrying *miR-200* for transfer to nonmetastatic cells, promoting less metastatic cells to distant metastasis (100). Dong et al. have demonstrated that BC cell-derived exosome *PTPRO* inhibits BC invasion and migration by promoting macrophage M1 polarization (101). Ham et al. have suggested that *Gp130* is a receptor for IL-6, which is present in BC cell-derived exosomes, and it promotes macrophage polarization to M2 and the formation of a cancer immunosuppressive microenvironment, thereby promoting cancer development (102).Zhou and colleagues have revealed that in the early stage of BC, the content of *miR-105* in cancer and circulation is closely related to the expression of the targeted tight junction protein ZO-1 and the metastatic progression of cancer, which is mainly achieved by downregulating the tight junction and destroying the barrier function of the endothelial cell monolayer (36). A previous study by Guo et al. has shown that BC cell-derived exosomes transport *miR-20a-5p* to promote osteoclast formation and thereby participate in bone metastasis of BC cells (103). Even in triple-negative breast cancer, Li et al. have shown that exosomes loaded with PD-L1 secreted by BC cells can inhibit the TIME and promote the progression of triple-negative breast cancer (104).

#### 2.2.4 Prostate cancer

Exosomes are involved in the occurrence and progression of prostate cancer. Wang and colleagues have revealed that the coculture of exosomes secreted by low-grade prostate cancer cells with highly malignant prostate cancer cells inhibits the proliferation, migration, and invasion of highly malignant cells (105). Singh et al. have demonstrated that exosomes derived from metastatic prostate cancer transfer αvβ3 integrin, distorting the function of non-cancer cells and promoting their migratory phenotype (106). Li and coworkers have reported that exosomes derived from prostate cancer can increase the expression of *CXCR4* in MDSCs by activating the *TLR2*/NF-κB pathway, thereby promoting the migration of MDSCs to the TIME and enhancing the formation of a cancer immunosuppressive microenvironment (107). Zou et al. have reported that prostate cancer-derived exosomal *miR-1275* promotes osteoblast activity and may be involved in cancer bone metastasis (108).

#### 2.2.5 Glioma

Exosomes are involved in the occurrence and progression of glioma. Glioma is cancer originating from glial cells. Experiments have shown that exosomes secreted by glioma-associated stem cells increase the invasiveness of GSSCs and GBM cell lines *in vitro*, which is related to *Semaphorin7A* (*SEMA7A*) in glioma-associated stem cells (109). Li et al. have demonstrated that *circGLIS3* (hsa_circ_0002874, originating from exon 2 of *GLIS3*) in exosomes promotes high-grade glioma invasion by regulating *Ezrin* phosphorylation (110).Wang Xu and colleagues have revealed that TDEs of glioblastoma (GBM) can fuse to the surface of T cells, increase the concentration of adenosine around T cells, activate *adenosine receptor 2A (A_2A_R*), and inhibit aerobic glycolysis in T cells, thereby suppressing T-cell clonal proliferation by reducing energy production. It promotes the formation of a cancer immunosuppressive microenvironment (111). Acquired drug resistance restricts the conventional treatment of gliomas. Zhang and colleagues have found that *lncRNA SBF2 antisense RNA 1* (*lncRNA SBF2-AS1*)-enriched exosomes are involved in temozolomide resistance of thermoresponsive GBM (112).

#### 2.2.6 Lung cancer

Exosomes are involved in the occurrence and progression of lung cancer. Lung cancer is one of the malignant cancers with the fastest increasing morbidity and mortality. Mao et al. have reported that *miR-2682-5p* in non-small cell lung cancer (NSCLC) exosomes can inhibit the viability and migration of NSCLC cells and promote cell apoptosis through the *HDAC1/ADH1A* axis (113). Chen et al. have recently demonstrated that c*ircular ubiquitin-specific protease-7 (circUSP7)* in NSCLC TDEs inhibits the secretion of IFN-γ, TNF-α, granzyme-b, and perforin by CD8+T cells and promotes the formation of a cancer immunosuppressive microenvironment (114). Liu et al. have pointed out that lung TDEs can promote the formation of an immunosuppressive microenvironment by enhancing M2 polarization, leading to cancer progression (115).

#### 2.2.7 Other cancers

Hou and coworkers have reported that salivary gland adenoid cystic carcinoma (SACC)-derived exosomes can act on human umbilical vein endothelial cells to promote cancer invasion (116). Ambrosini et al. have reported that uveal melanoma exosomes promote cancer progression by inducing a cancer immunosuppressive microenvironment through macrophage *migration inhibitory factor (MIF*) *(* 117). Chen et al. have demonstrated that Nasopharyngeal carcinoma-derived exosomes transfer the macrophage *MIF*, inhibit macrophage ferroptosis, and promote M2 polarization (118). Xia et al. have indicated that exosomes derived from clear cell renal cell carcinoma evade innate immune surveillance by regulating the TGF-β/*SMAD* pathway, thereby inducing NK-cell dysfunction (119).

## 3 Exosome immunotherapeutic strategy for malignant cancers

**Figure 3:**
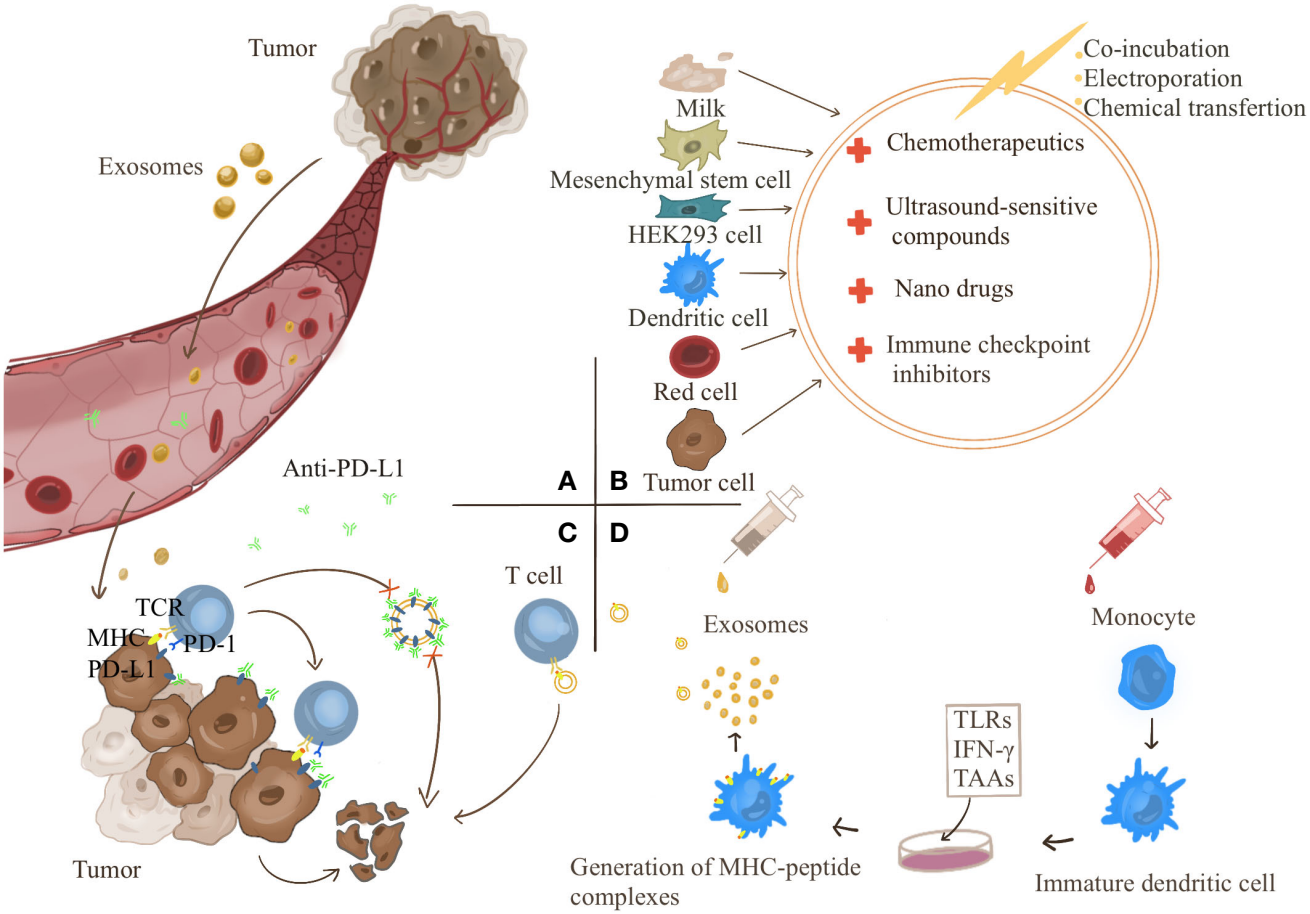
**(A)** Tumor-derived exosomes can act directly on tumors or be transported to the tumor site through the blood to cause tumor development. Targeting the targets produced by exosomes is a strategy for tumor treatment. **(B)** Exosomes have become good carriers for drug delivery due to their good biocompatibility. Exosomes can be derived from milk, mesenchymal stem cells, HEK293 cells, dendritic cells, red blood cells, cancer cells, etc. Anticancer drugs can be transferred into exosomes by co-incubation, electroporation, and chemical transfection. **(C)** Exosomes competitively consume PD-L1 antibody and participate in the resistance of PD-L1 antibody treatment. **(D)** Therapeutic strategies for exosome vaccines. Monocytes were induced to differentiate into immature DC cells *in vitro* and then treated with the corresponding tumor-associated antigens (TAAs), Toll-Like Receptors (TLRs) and chemokines such as IFN-γ make DC cells differentiate and mature and secrete exosomes carrying specific tumor antigens, which can directly activate the body’s immunity.

Exosomes provide a therapeutic strategy for cancer immunotherapy (**Figure 3**). Exosomes are involved in the formation of cancer immunosuppressive microenvironment. Therefore, targeting the production process of tumor exosomes may become an effective treatment modality.As an inert biocompatible substance, exosomes can exert their superior drug delivery advantages to treat cancers. In addition, exosomes also play an essential role in the high-profile immune checkpoint inhibitor PD-1/PD-L1 treatment. Finally, TDEs carry information from primary cells and express complexes of MHC class I/II epitopes and costimulatory molecules, which provide novel strategies for cancer vaccines.

### 3.1 Therapeutic strategies targeting exosome production

As mentioned above, exosomes contain various small molecular substances that mediate the occurrence and development of cancers. Therefore, targeting the production of exosomes is a strategy to increase or decrease certain small molecular substances to affect the development of cancers. Guan et al. have indicated that high-dose Proton-pump inhibitors (PPIs) suppress cancer size in cancer-bearing BALB/c nude mice, most likely mediated by inhibiting exosome release through suppressing ATP production (124). Huang and colleagues have found that the anticancer effect of lecithin is mediated by the reduction of exosome production and thus the downregulation of *miR-1246* carried by them (125). Wang and colleagues have revealed that *antigen-presenting cell (APC)-activated lncRNA (lncrNA-APC1)* can directly regulate the stability of *Rab5b* mRNA to inhibit the production of exosomes in CRC cells, thereby suppressing angiogenesis and limiting cancer growth and proliferation (126). Similarly, promoting the secretion of exosomes can also achieve some anticancer effects. Xu et al. have indicated that hyperthermia can promote the secretion of exosomes through *Rab7b*, upregulate the transfer of *FOS* and *CREB5* anticancer genes into drug-resistant cancer cells, and increase the sensitivity of BC cells to doxorubicin (Dox) (127). All these studies provide potential therapeutic targets. The *RAB27A*-targeting inhibitor tipifarnib (128), sphingomyelinase inhibitor GW4869 (129), manumycin A (130), and other drugs have been developed, and the clinical safety and efficacy of these drugs need to be further studied.

### 3.2 Exosomes for targeted drug delivery

Exosomes can be loaded with both small and large molecules, supporting their use as therapeutic tools for treating various diseases, including cancer (131). The construction of engineered exosomes loaded with drugs provides a more efficient, less toxic and more targeted drug delivery strategy (132, 133). It has attracted attention due to its good biocompatibility (134, 135). Exosomes can be derived from milk, MSCs, HEK293 cells, DCs, red blood cells, and cancer cells (136). Agrawal et al. have suggested that milk-derived exosomes used for oral delivery of paclitaxel exhibit more marked inhibitory effects *in vivo* (137). Kanchanapally and colleagues have found that macrophage-derived adriamycin (DOX)-loaded exosomes are more cytotoxic than TDEs (132). This finding provides a basis for selecting raw materials for engineered exosomes. In addition, exosomes carry different peptides, tetraspanins and integrins to target specific cells, which provides strategies for the construction of more specific drug delivery (138–141). Cui et al. used a bone-targeting peptide to modify exosomes to deliver siShn3, which achieved the anti-osteoporosis effect of targeting osteoblasts (142). Compared with liposomes, exosomes have a stronger targeting effect, mainly because CD47 has the ability to evade the phagocytosis of mononuclear macrophages, which was confirmed in the study of Sushrut Kamerkar (143). Therefore, it is not difficult to think that artificial modification of exosomes can better enhance their targeting ability. Zhang et al. integrated hybrid membrane proteins from red blood cells and MCF-7 cancer cells into exosomes and designed an artificial chimeric exosome. Membrane proteins from red blood cells contain high CD47 levels and can inhibit phagocytosis, while membrane proteins from MCF-7 cancer cells contain specific adhesion proteins that can adhere to homologous cancer cells. This exosome obtained significant anti-cancer ability in mouse experiments (144). In addition, Qi et al. shown that superparamagnetic nanoparticle modified exosomes enhanced cancer targeting under external magnetic field and suppressed liver cancer in mice (145). Therefore, exosomes are highly valuable drug tools.

The exosome carrier strategy can also be combined with sonodynamic therapy and nanomedicine to give full play to the advantages of both sides. Wang et al. have developed Exo^Ce6+R848^ by coincubating ultrasound-sensitive Chlorin e6 (Ce6) and R848 into exosomal membranes. Under ultrasound, EXO^Ce6+R848^ stimulates robust maturation of cancer-infiltrating DCs, promotes M1 macrophage polarization and proinflammatory cytokine production, and decreases anti-inflammatory cytokines. This process further inhibits Tregs in the TIME and activates effector T cells for anticancer purposes (146). Although much attention has been paid to drugs, such as nanomedicines (147, 148), the exosome carrier strategy has attracted much attention due to controversial toxicity, immune rejection, and stability (149). Wu and colleagues have used catalase (CAT) and the sonosensitive agent indocyanine green-loaded silica nanoparticles (CAT@SiO2), which is known as a biodegradable nanoplatform (termed as CSI), to coat macrophage exosomes with CSI to form CSI@Ex-A. In addition, exosomes overcome the blood-brain barrier obstruction of the original CSI, making it more effective in clinical practice (150). Various methods exist to transfer anticancer drugs into exosomes, including coincubation, electroporation, and chemical transfection (151, 152) However, the transfer efficiency of multiple processes is different, and exploring more effective construction strategies requires more in-depth research.

Dual-targeting exosome immunotherapy based on exosome delivery to activate the immune system and inhibit immune checkpoints is a more effective emerging strategy. Fan et al. have suggested that by using a dual-targeting exosome-loaded drug, cGAMP (cGAMP@dual-anti-Exos), whose surface is modified with two antibodies (anti-PD-L1 and anti-CD40), two-step activation of DCs and blocking of cancer cell PD-L1, the survival rate of mice inoculated with the drug is up to 60%. In contrast, all other mice die after 40 days, indicating that the drug significantly improves cancer suppression (153). Wang et al. have used the homing ability of cancer cells and macrophages to develop a chimera that secretes outside the body. The cancer cell nucleus embedded within macrophages causes the secretion of macrophage cancer signals to carry body secretion. It relies on the homing ability and lymph nodes to achieve double targets, activates the body’s immune system, removes cancer immunosuppression, and increases anticancer ability. When combined with the immune checkpoint inhibitor PD-1 treatment, chimeric exosomes significantly prolong the survival of a mouse model of cancer recurrence (154).

### 3.3 Exosomes and PD-1/PD-L1

PD-1/PD-L1 is a crucial target of immune checkpoint inhibitor therapy. PD-L1 in TDEs can induce PD-L1 expression on immune cells, such as neutrophils, inhibit T-cell activation, and promote cancer growth (78, 155). In immunotherapy, exosomes are involved in resistance to immune checkpoint inhibitor therapy targeting PD-L1. One possible mechanism is that exosomes deplete PD-L1 antibodies and increase resistance to PD-L1 therapy (156). In a study by Mauro and collaborators, anti-PD-L1 antibodies synergize with exosomal PD-L1 blockers to inhibit cancer growth. This finding suggests that exosomal PD-L1 is a novel target for relieving cancer drug resistance (45). Various strategies have been developed based on the above mechanisms to target exosomal PD-L1. Blocking any step in exosome production can reduce exosomal PD-L1 expression (157). Therefore, drugs that block exosome production can reduce exosome PD-L1. Shen et al. have shown that blockade of the *LSD1* target specifically reduces exosomal PD-L1 expression without reducing intracellular PD-L1 expression and activates T-cell killing, providing a more precise choice of targets for inhibiting exosomal PD-L1 expression (158). In addition, increasing the elimination of exosomes can be therapeutic, such as *in vitro* ultrafiltration (159), while this approach also removes exosomes from normal cells, and its safety needs to be studied. Wang Min et al. have demonstrated that lactadherin promotes the clearance of circulating cancer cell-derived EVs, which also provides an exosome clearance mode (87). Direct inhibition of PD-L1 production is also a strategy. Shin et al. have reported that sulfamethoxazole effectively reduces exosomal PD-L1 content, thus allowing the anti-PD-L1 antibody to exert a potent anticancer effect. In addition, because inhibition of exosomal PD-L1 enhances T-cell immunity, it also increases the response rate to anti-PD-1 antibodies (160).

### 3.4 Cancer vaccines

Exosomes have the potential to be used as cancer vaccines. As carriers of information transmission, exosomes carry information about the original cell and express complexes of MHC class I/II epitopes and costimulatory molecules. Therefore, they can also activate CD8 T cells. Moreover, exosomes have good biological stability and bioavailability, providing a basis for their application in cancer vaccines (44, 161–163). As early as 2004, Altieri and coworkers have reported that plasmacytoma cells release exosomes *in vitro*, and a single dose (5 µg) of exosomal protein combined with an isogenic vaccine can protect 80% of mice against wild-type cancer challenge, making exosome vaccines possible (164). There are two significant obstacles to the success of DC vaccines: cancer-mediated immunosuppression and the functional limitations of commonly used monocyte-derived DCs, which are effectively avoided by using cancer cell exosomes (165). It has been found that vaccination with DCexos (derived from BMDCs) has better anticancer efficacy than vaccination with DCs (44, 166).

Exosomes can be designed as vaccine adjuvants. Yildirim and colleagues have developed BC cell-derived exosomes carrying two immune adjuvants, the TLR9 ligand, K-type CpG ODN and a TLR3 ligand, p(I:C), to achieve more vigorous cellular and humoral anticancer immunity. In addition, it can inhibit cancer growth in mice (167). Experiments have shown that transfer of *CCL22* siRNA into immunologically dead TDEs can inhibit Treg expansion and enhance anticancer effects. Better anticancer efficacy has been achieved when combined with chemotherapy drugs (168). Exosomes derived from M1-polarized macrophages can induce the release of a Th1 cytokine repertoire and antigen-specific cytotoxic T-cell responses, acting as immunopotentiating agents for cancer vaccines (169), most likely through downregulation of *Wnt* signaling (170). Kavitha et al. have demonstrated that exosomes from mouse embryonic stem cells engineered to produce *GM-CSF* activate a higher CD8+ T effector, Th1-cell response, and significantly block cancer growth (171). Huang et al. have developed an exosome loaded with Hiltonol (*TLR3* agonist) and ICD (immunogenic cell death) inducer human neutrophil elastase (*ELANE*). In addition, the engineered Hiltonol-elane-α-la exosomes (HELA-Exos) are modified with α-lactalbumin on the exosome surface, which induces strong anticancer immunity (172). Phung and colleagues combined immune checkpoint blockade strategies with cancer vaccines, and modified anti-CTLA-4 antibodies on the surface of exosomes by lipid anchoring, which effectively promoted T cell targeting and increased tumor-specific T cell responses, leading to significant tumor growth inhibition (173). Modifying exosomes with MART-1 peptides to induce a stronger immune response (168). These strategies show the potential of exosomes as cancer vaccines to specifically activate anti-tumor immunity.

## 4 Conclusions and future perspectives

This article reviews the research progress of exosomes, mainly taking gastrointestinal cancers as an example, and introduces the research progress of exosomes in cancers and cancer immunotherapy. Clearly, understanding the mechanism of action of cancer exosomes in cancers provides new options for therapeutic targets, as well as promising biomarkers for the delivery of exosome-loaded small molecules that can be engineered for less invasive cancer diagnosis (174). However, the discovery of more efficient exosome isolation methods is a challenge (112). Chen et al. have explored a microfluidic device for on-chip isolation for immunomagnetic separation and *in situ* detection of blood exosomes. The device also has a high prediction accuracy for cancer extracellular markers, with a sensitivity of 90% and a specificity of 95% (175). Furthermore, Thakur and colleagues have developed an in-house novel exosome detection/separation strategy and a localized surface plasmon resonance biosensor with self-assembled gold nanoislands (SAM-AuNIs) (176). This finding provides a method for us to make better use of exosomes.

There is still a long way to go for exosome-related research. Previous studies have shown that cancer cell chromosomal DNA fragments can be found in TDEs, while the specific sorting mechanism has not been determined (177). Moreover, some of the studies reviewed in this paper are limited to *in vitro* experiments, and the data and conclusions *in vivo* are likely to be affected by environmental interactions. Therefore, its guidance for clinical practice is limited, and more studies are needed to support it. In addition, most studies at this stage are limited to small nucleic acids and proteins, and the role of lipid components has also attracted much attention in recent years (178). In cancer treatment, exosomes can be designed as carriers or targets to develop therapeutic strategies to relieve therapeutic drug resistance. As potential vectors for dual-targeted therapy, *ARG1* (179), *TIM3*and *EBAG9* (180) have been confirmed to exist in exosomes, while there are few studies, and their more precise regulatory mechanisms need to be further studied. In conclusion, although significant progress has been made in the analysis of exosomes, many issues still need to be studied further. These include better strategies for sorting exosomes carrying genetic materials, improved extraction and detection methods, and more stable and accurate strategies for modifying drugs delivered *via* exosomes. Finally, exosomes show great promise as cancer immunotherapy. As knowledge grows, therapeutic approaches based on exosomes will become increasingly used in clinical settings.

## Author contributions

YL and WG designed and conceptualized the review. YC and YL wrote the manuscript. WG and YL revised the manuscript. All authors contributed to the article and approved the submitted version.
